# Temperature Dependence of Single Step Hydrodeoxygenation of Liquid Phase Pyrolysis Oil

**DOI:** 10.3389/fchem.2018.00297

**Published:** 2018-07-19

**Authors:** Klara Treusch, Nikolaus Schwaiger, Klaus Schlackl, Roland Nagl, Peter Pucher, Matthäus Siebenhofer

**Affiliations:** ^1^BDI – BioEnergy International GmbH, Research and Development, Raaba-Grambach, Austria; ^2^Institute of Chemical Engineering and Environmental Technology, Graz University of Technology, Graz, Austria

**Keywords:** hydrodeoxygenation, liquid phase pyrolysis, pyrolysis oil, temperature variation, 2^nd^ generation biofuels

## Abstract

In this paper, continuous hydrodeoxygenation (HDO) of liquid phase pyrolysis (LPP) oil in lab-scale is discussed. Pyrolysis oil is derived from the bioCRACK pilot plant from BDI - BioEnergy International GmbH at the OMV refinery in Vienna/Schwechat. Three hydrodeoxygenation temperature set points at 350, 375, and 400°C were investigated. Liquid hourly space velocity (LHSV) was 0.5 h^−1^. Hydrodeoxygenation was performed with an *in situ* sulfided metal oxide catalyst. During HDO, three product phases were collected. A gaseous phase, an aqueous phase and a hydrocarbon phase. Experiment duration was 36 h at 350 and 375°C and 27.5 h at 400°C in steady state operation mode. Water content of the hydrocarbon phase was reduced to below 0.05 wt.%. The water content of the aqueous phase was between 96.9 and 99.9 wt.%, indicating effective hydrodeoxygenation. The most promising results, concerning the rate of hydrodeoxygenation, were achieved at 400°C. After 36/27.5 h of experiment, catalyst deactivation was observed.

## Introduction

Biomass pyrolysis is a suitable pathway for the production of second generation biofuels (Demirbas, 2011). During pyrolysis, one of the major products is pyrolysis oil. Due to its high water content, high corrosivity and other negative properties, according to Table 1, pyrolysis oil needs intensive upgrading prior to usage as fuel for combustion engines. To achieve fuel quality standards, an upgrading step is necessary. Hydrodeoxygenation (HDO) is a high potential upgrading technology (Pucher et al., 2015). In literature, mainly experiments with fast pyrolysis oil are reported.

**Table 1:** Properties and composition of LPP oil.

**Property**	**Unit**	**LPP oil**
Water content	[wt.%]	57.0
Lower heating value	[MJ/kg]	7.4
Density	[kg/m3]	1092
Viscosity	[mPa·s]	3.5
Carbon content	[wt.%]	22.3
Hydrogen content	[wt.%]	9.4
Oxygen content (balance)	[wt.%]	67.8
Nitrogen content	[wt.%]	< 1

One of the biggest issues during HDO of pyrolysis oil in general is catalyst deactivation caused by coke formation. Especially the single-step HDO above 300°C is seen as troublesome, as it leads to coking and plugging (Elliott and Bager, 1989). Therefore, a two-step process is proposed in literature (Elliott, 2007; Elliott et al., 2009; Carpenter et al., 2016; Meyer et al., 2016; Olarte et al., 2016, 2017). In a first step, pyrolysis oil is stabilized (Pucher et al., 2014) through mild hydrotreatment at low temperature. In a second step, the final hydrodeoxygenation, or hydrocracking, takes place. Hydrotreatment temperatures are between 140 and 375°C, at liquid hourly space velocities between 0.28 and 0.5 h^−1^. The hydrocracking step is performed at temperatures of about 400°C and liquid hourly space velocities of 0.1–0.4 h^−1^ (Elliott et al., 2009; Olarte et al., 2016).

Contrary to these results, in this paper LPP oil is processed continuously in a single-step HDO reactor at 350–400°C. The LHSV was set on the limits of HDO of fast pyrolysis oil with 0.5 h^−1^ (Volume LPP oil/ Volume of empty tube and hour).

## Liquid phase pyrolysis

In liquid phase pyrolysis, biomass is pyrolyzed in a liquid heat carrier (Schwaiger et al., 2011, 2012). During this conversion, a part of the biomass dissolves in the heat carrier, while a second liquid phase, a polar water containing hydrocarbon phase, is generated (Schwaiger et al., 2015). In the bioCRACK process (Ritzberger et al., 2014; Treusch et al., 2017), LPP was operated with the heat carrier vacuum gas oil to enable integration in an oil refinery. From 2012 to 2014 a pilot plant was operated by BDI – BioEnergy International GmbH at the OMV refinery in Vienna/Schwechat.

## Materials and methods

Experiments were carried out in a plug flow reactor with an inner diameter of 3/8 inches and a heated zone of about 30 cm, made by Parr Instrument Company. It was designed for a maximum pressure of 220 bar and a maximum temperature of 550°C. The temperature was detected by an inner thermowell with a thermocouple with three probe points. Heat was provided by a single zone external electric heater. In the temperature range between 350 and 400°C, three operation points were tested: 350, 375, and 400°C. Hydrogen pressure was kept constant at 121.5 bar for all experiments.

### Materials

The LPP oil was derived from the bioCRACK pilot plant. It was produced by LPP of spruce wood. The composition of LPP oil is shown in Table 1.

HDO was performed with a sulfided CoMo/Al_2_O_3_ catalyst, details are shown in Table 2. It was obtained as extrudates with a length of 2–3 mm. The catalyst was chosen as it is cheaper than noble metal catalysts and not susceptible for catalyst poisoning through sulfur, in contrary it gets more active by adding sulfur. For sulfidation, 35 wt.% di-tert-butyldisulfide (DTBDS) in decane was used. To provide enough sulfur during HDO, 150 ppm of sulfur as DTBDS were added to the LPP oil. Hydrogen 5.0 was provided in a 300 bar gas cylinder from AIR LIQUIDE AUSTRIA GmbH.

**Table 2:** Catalyst details (CoMo/Al_2_O_3_).

**Supplier**	**Alfa Aesar**
Cobalt oxide [wt.%]	4.4
Molybdenum oxide [wt.%]	11.9
Surface area [m^2^/g]	279
Stock number	45579

### Analytical methods

The ultimate analysis of all streams was done by a vario MACRO CHN-analyser from Elementar Analysensysteme GmbH. The oxygen was determined by difference. The water content of the aqueous product phase was determined by a gas-phase chromatograph, type Agilent 7890A, with a TCD-detector and a HP-INNOWAX column, 30 m^*^0.530 mm^*^1 μm. For determination of the water content, the GC was calibrated with high-purity water (type I) in THF in the range of 1–8 wt.% water. The boiling range of the hydrocarbon product phase was determined by a gas-phase chromatograph, type Agilent 7890A, with a FID-detector and a Restek-column MXT-2887, 10 m ^*^0.530 mm ^*^2.65 μm, according to ASTM Method D2887. The water content of the oil fraction was determined by Karl-Fischer-titration with a Schott Titro Line KF-Titrator and a Hydranal titration reagent. Density and viscosity were measured by a digital viscosimeter, SVM 3000, of Anton Paar GmbH. The composition of the hydrocarbon product phase was determined by gas chromatography-MS with a quadrupole mass spectrometer (GC-MS), type Schimadzu GCMS QP 2010 Plus, with a VF-1701 MS column, 60 m ^*^0.25 mm ^*^0.25 μm. The GC-MS was calibrated with a multi-component standard, consisting of: pentane, 2-methyl-pentane, hexane, methyl-cyclohexane, ethyl-cyclopentane, octane, toluene, ethyl-cyclohexane, propyl-cyclohexane and decane in THF, in the range of 100–3,000 ppmw each. Additionally, flouranthene was used as internal standard. The gas phase composition was determined by a micro gas-phase chromatograph (micro-GC), type Agilent 3000A, with a TCD-detector, a molecular sieve column and a plot u column. The micro-GC was calibrated with oxygen, nitrogen, hydrogen, methane, ethane, acetylene and carbon dioxide.

### Catalyst preparation

To increase the specific surface area, the catalyst was milled in a centrifugal mill with trapezoidal perforations of 1 mm diameter. The ground material was sieved in a sieving tower of Retsch to obtain the target particle size of 200–600 μm. The reactor was then filled upside down with catalyst. On bottom and top a few cm of catalyst extrudates were applied. The heated zone of the reactor (30 cm) was filled with particles of 200–600 μm size. The catalyst was held in the reactor with a sieve at the bottom.

For each experiment, the reactor was filled with fresh CoMo/Al_2_O_3_ catalyst of Alfa Aesar and inertised with nitrogen. Afterwards the reactor was flushed with hydrogen. Then a hydrogen flow rate of 0.5 l/h was adjusted.

For the activation of the catalyst, a sulfidation step preceded the HDO experiments. Thus, 35 wt.% di-tert-butyldisulfide (DTBDS) in decane was pumped through the reactor during heating up. Sulfidation was continued for five hours at 400°C. After sulfidation, the temperature was reduced to the requested temperature of HDO procedure.

### Experimental procedure

After sulfidation, 5 h of HDO of LPP oil were performed in the unsteady state operation mode. Afterwards, 36 h of HDO were performed, with liquid product sampling every 12 h for experiments at 350 and 375°C. At 400°C, experiment duration was 27,5 h and sampling periods were 8, 7.5, and 12 h. The gas phase composition was monitored every 4 h. After 36/27.5 h of steady state operation, the reactor was shut down and the catalyst bed was washed with acetone for catalyst analysis. Plugging was not observed.

## Results

In this chapter, observations during experiments, mass balance and product characterization are given. Possible pathways of biomass constituents to components in the final product, derived from GC-MS analysis, are discussed.

### Temperature profile in the reactor

**Figure 1:**
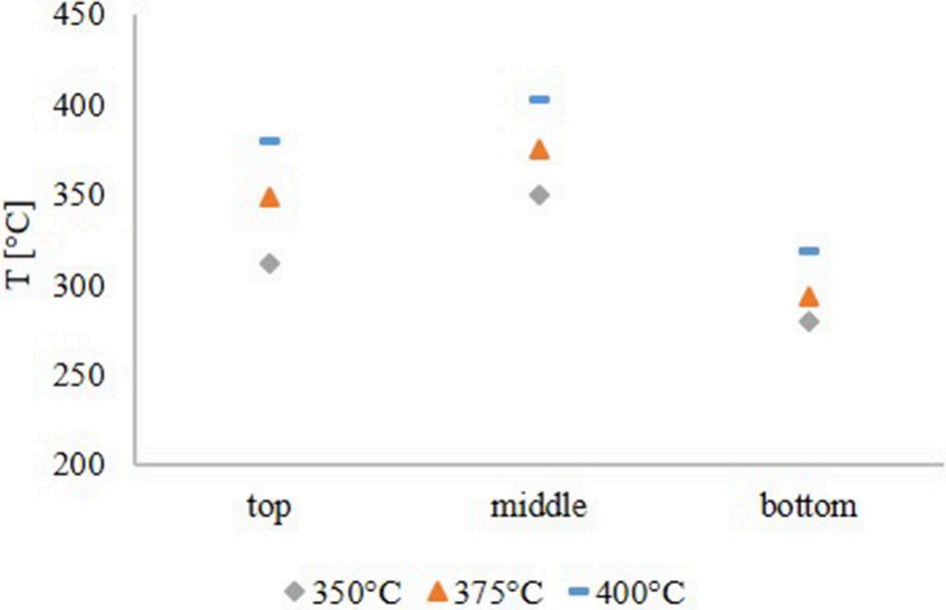
Temperature profile in the reactor.

The temperature profile, shown in Figure 1, was similar for all experiments. Due to the fact, that the pyrolysis oil was not pre-heated, the feed temperature was lower than the temperature in the middle of the reactor. The temperature maximum was obtained in the middle of the reactor. This temperature was the set point temperature for all HDO experiments. At the exit of the reactor, the temperature dropped significantly due to external cooling effects. From the temperature profile it was concluded, that the exothermal HDO reaction was completed after about 2/3 of the reactor.

#### Mass balance and coke formation

During HDO, three product phases were formed. A hydrocarbon phase, the target product, an aqueous phase and a gaseous phase. In general, the difference concerning the product distribution between hydrocarbon product and aqueous phase is not depending on temperature in the range of 350–400°C. The differences are more recognizable in the stream compositions. Table 3 shows that the yield of aqueous phase decreased with temperature, whereas the gas yield increased.

**Table 3:** Mass balance based on LPP oil and H_2_ feed.

**Temperature**	**350^°^C**	**375^°^C**	**400^°^C**
LPP oil [wt.%]	79.13	79.32	80.47
H_2_ [wt.%]	20.87	20.68	19.53
Aqueous [wt.%]	59.96	58.94	58.62
Hydrocarbon [wt.%]	7.68	7.76	7.79
Gaseous [wt.%]	26.82	27.55	28.37
Coke [wt.%]	1.34	1.35	1.36

**Figure 2:**
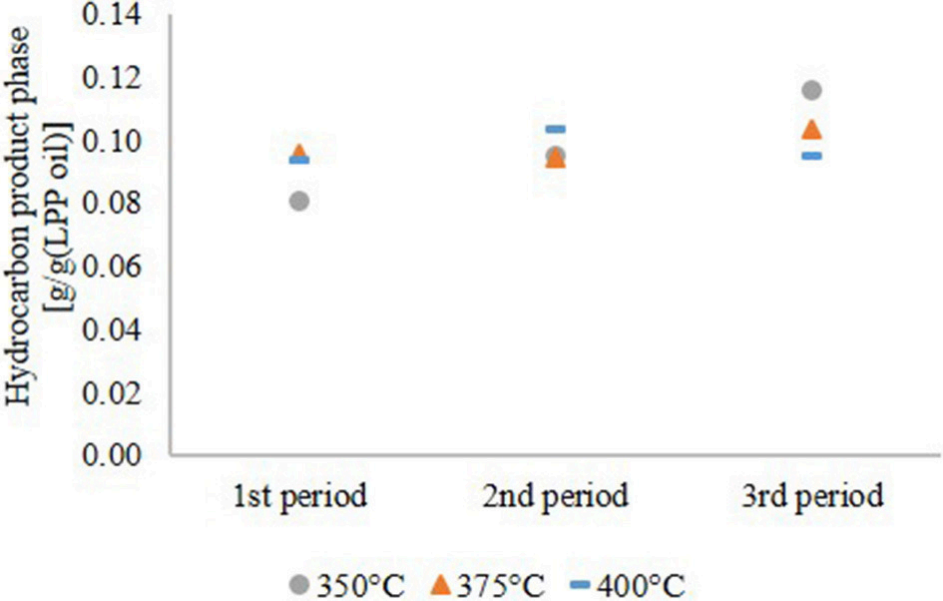
Yield on hydrocarbon product phase based on LPP oil.

The yield of the hydrocarbon product phase based on the LPP oil in the feed is shown in Figure 2. At 350 and 375°C it increased continuously until the end of experiment. The increasing production rate of organic phase at 350 and 375°C is not caused by a higher conversion of LPP oil to fuel, it is rather a consequence of incomplete HDO. This indicates faster catalyst deactivation at lower temperatures. However, at 400°C the hydrocarbon product yield was constant.

#### Rate of HDO

**Figure 3:**
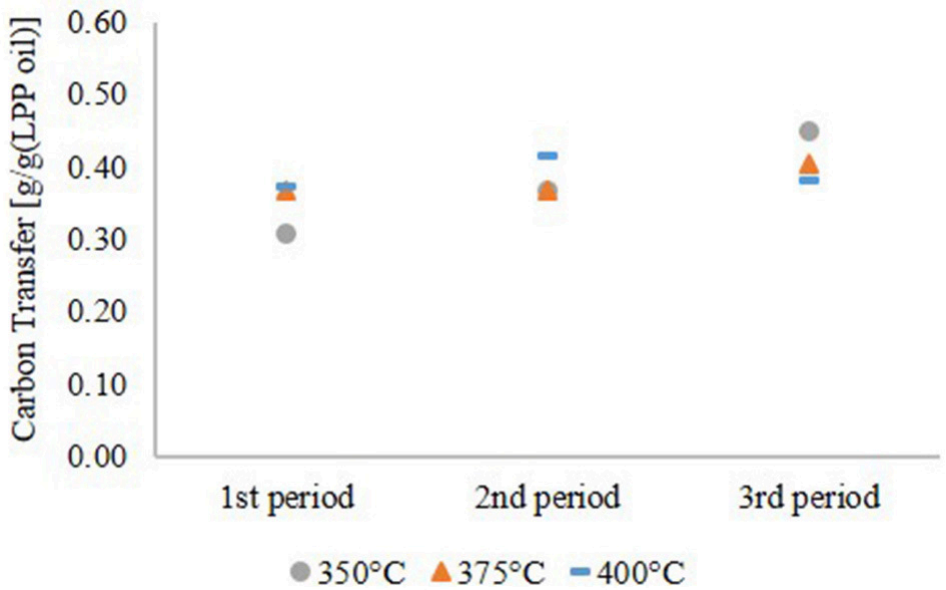
Carbon transfer into the hydrocarbon product phase.

As shown in Table 1, LPP oil contains a high amount of water. Yield, based on the LPP oil feed, was therefore low too. Referring to the carbon content of LPP oil, a carbon transfer into the hydrocarbon product phase, given in Figure 3, of up to 45 wt.% was obtained. The rest merged into the gas phase. Scattered carbon transfer was observed at 350°C HDO temperature. After 12 h of operation, it was only about 30 wt.% and increased to 45 wt.% after 36 h. This observation goes along with the yield of hydrocarbon products and is partly caused by a higher oxygen content. This leads to the conclusion, that more polar compounds are dissolved in the hydrocarbon product phase. Due to the lower gas yield one can also assume, that less cracking reactions occurred due to deactivation of the catalyst at low temperature. The carbon transfer increased slightly at 375°C and was nearly constant at 400°C, indicating stable catalyst performance.

The H/C ratio, is a very significant indicator for characterizing the degree of hydrogenation. In combination with the oxygen content, it quantifies the degree of HDO. The oxygen content was derived from the balance of the ultimate analysis. According to Table 4, the oxygen content was zero for all experiments over the whole time range except for the experiment at 350°C after 36 h of operation. It is obvious that at this temperature the activity of the catalyst depleted during the experiment. Therefore, the H/C ratio can be considered as main quality criterion for the degree of HDO for all other data points.

**Table 4:** Oxygen content of the organic product phase (determined by balance of the ultimate analysis).

**Oxygen [wt.%]**	**1^st^ Period**	**2^nd^ Period**	**3^rd^ Period**
350°C	0.00	0.00	1.11
375°C	0.00	0.00	0.00
400°C	0.00	0.00	0.00

**Figure 4:**
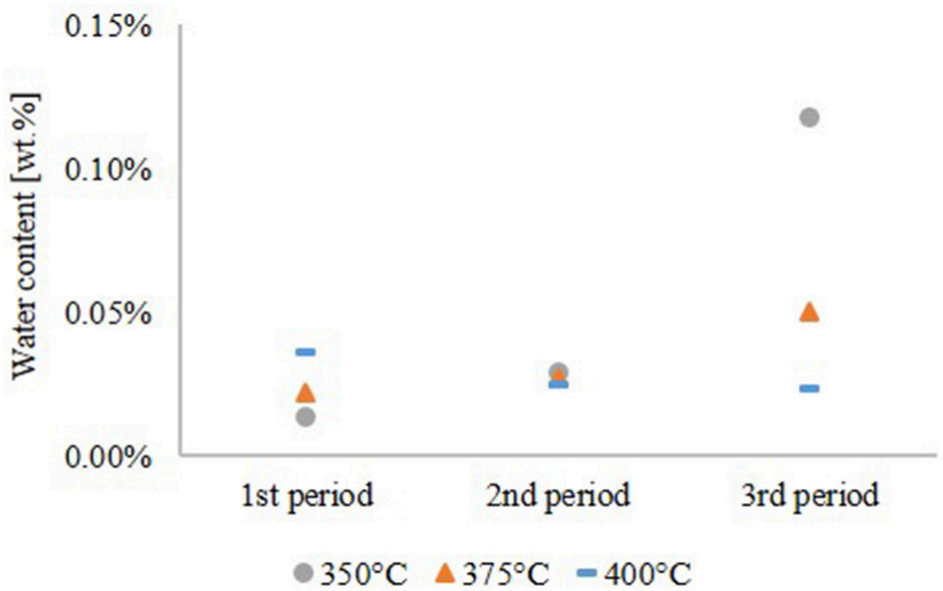
Water content of the hydrocarbon product phase.

As shown in Figure 4, the water content of the hydrocarbon product phase correlates with the oxygen content. Except the experiment at 350°C, the water content of the hydrocarbon product phase was 0.02–0.05 wt.%.

**Figure 5:**
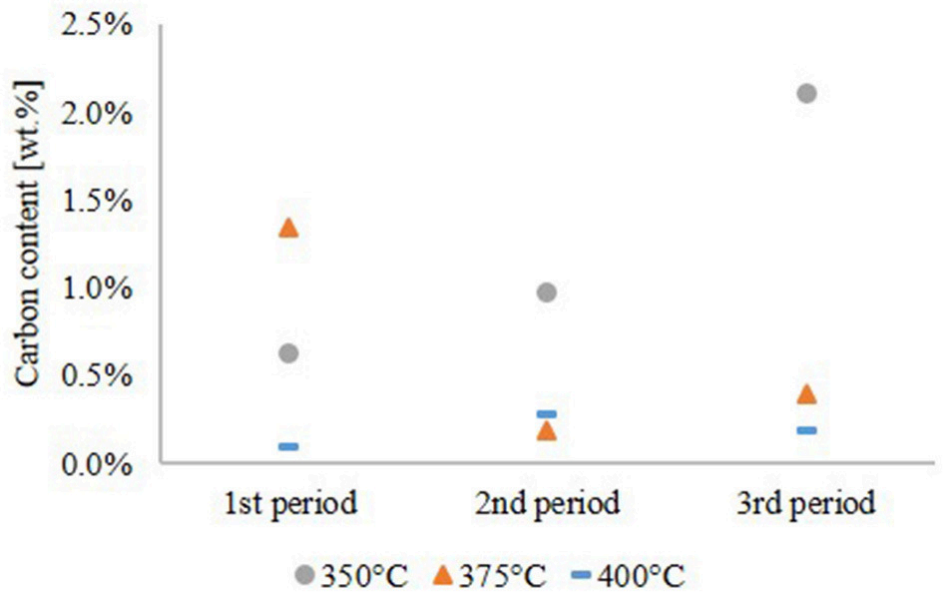
Carbon content of the aqueous phase.

The carbon content of the aqueous phase, given in Figure 5, is a complementary quality parameter for the HDO performance. A high carbon content of the aqueous phase correlates with a high oxygen content of the hydrocarbon product phase due to incomplete hydrophobation of LPP oil. At 350°C, the carbon content of the aqueous phase increased during the experiment with a maximum of about 2.1 wt.%. The opposite happened at 375°C, where the carbon content was highest in the first period of the experiment. At 400°C the carbon content, indicating carbon loss into the aqueous phase, was below 0.5 wt.% over the whole experiment and didn't show a trend.

The water content of the aqueous phase was between 96.9 and 99.9 wt.% in all cases, as shown in Table 5. This result confirms the low carbon loss into the aqueous phase and high effectiveness of HDO.

**Table 5:** Water content of the aqueous phase.

**Water [wt.%]**	**1^st^ Period**	**2^nd^ Period**	**3^rd^ Period**
350°C	99.9	99.7	97.8
375°C	97.0	97.6	96.9
400°C	98.5	97.6	98.1

**Figure 6:**
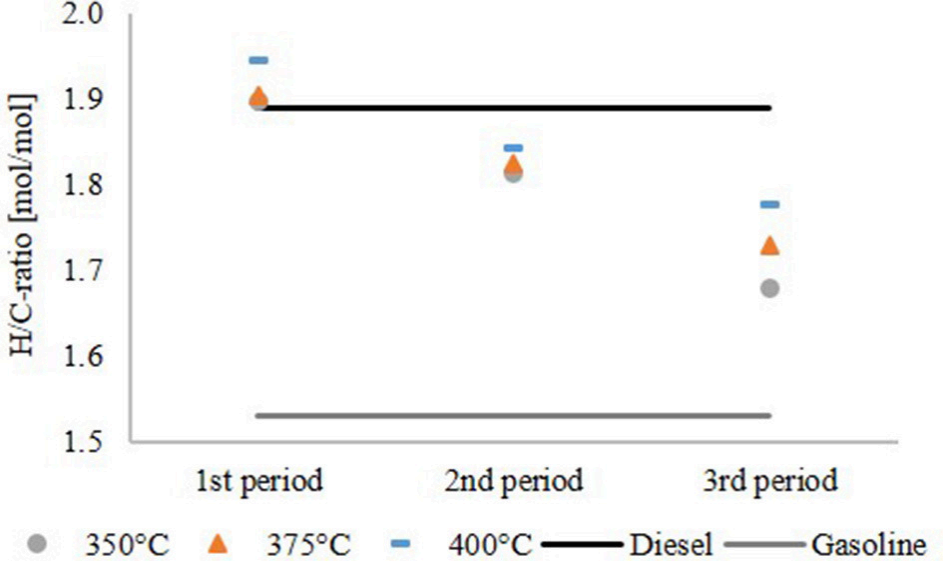
H/C ratio of the hydrocarbon product phase.

Figure 6 shows the H/C ratio of the hydrocarbon product phase compared to diesel and gasoline. For comparison, the H/C ratio of diesel with hydrotreated vegetable oil (HVO) additives and gasoline without biogenic additives were used. The H/C ratio decreased over the time span of the experiment and increased with the temperature. The highest H/C ratio was observed at 400°C in the first period of the experiment. Afterwards deactivation of the catalyst became detectable, although the H/C ratio was still in the range of diesel and gasoline. The results of the experiment at 350°C again confirmed a significant oxygen content, indicating insufficient HDO.

#### Product characterization

In Table 6, the properties of the hydrocarbon product phase, depending on the HDO temperature, are summarized and compared to diesel and gasoline. Water content, lower heating value, density, viscosity and boiling range are between the values for diesel and gasoline, indicating that the product is a mixture of diesel and gasoline and that these fractions can be obtained by distillation. Through the high grade of HDO a high heating value of about 42.7 wt.% was achieved.

**Table 6:** Hydrocarbon product characterization of the 2^nd^ period of experiment compared to diesel and gasoline.

**Compound**	**Unit**	**HDO 350^°^C**	**HDO 375^°^C**	**HDO 400^°^C**	**Diesel**	**Gasoline**
Water content	[wt.%]	0.03	0.03	0.03	< 0.02 EN 590, 2004	n.a.
Lower heating value (Boie; Grote and Feldhusen, 2007)	[MJ/kg]	42.68	42.73	42.72	43.2	41.8
Density	[kg/m3]	829	823	805	820–845 EN 590, 2004	720-775 EN 228, 2004
Viscosity	[mPa·s]	1.56	1.45	1.07	2.0–4.5 EN 590, 2004	n.a.
Boiling at 150°C	[V.%]	32.6	31.0	20.7	n.a.	≥75 EN 228, 2004
Boiling at 350°C	[V.%]	96.6	97.2	98.5	≥85 EN 590, 2004	n.a.
Carbon transfer	[%]	36.7	36.6	41.5	–	–
Carbon content	[wt.%]	86.37	86.35	86.03	86.3	88.7
Hydrogen content	[wt.%]	13.17	13.24	13.33	13.7	11.4
Balance (oxygen content)	[wt.%]	0.00	0.00	0.00	0.0	0.0
Nitrogen content	[wt.%]	< 1	< 1	< 1	< 1	< 1

The ultimate analysis is compared with gasoline without biogenic additives and diesel with HVO additives. The lower heating value was calculated with the algorithm of Boie (Grote and Feldhusen, 2007 Equation 1).

$$\begin{array}{rcl} LHV & = & 35\cdot c+94,3\cdot h-10,8\cdot o+10,4\cdot s \end{array}$$

(1) $$\begin{array}{r} +6,3\cdot n-2,44\cdot w \end{array}$$

with, *c, h, o, s, n* and *w* representing the amount of carbon, hydrogen, oxygen, sulfur, nitrogen and water in wt.%, respectively.

Water content, density, viscosity and boiling cut points of diesel and gasoline are derived from the standard of diesel (EN 590, 2004) and gasoline (EN 228, 2004).

**Figure 7:**
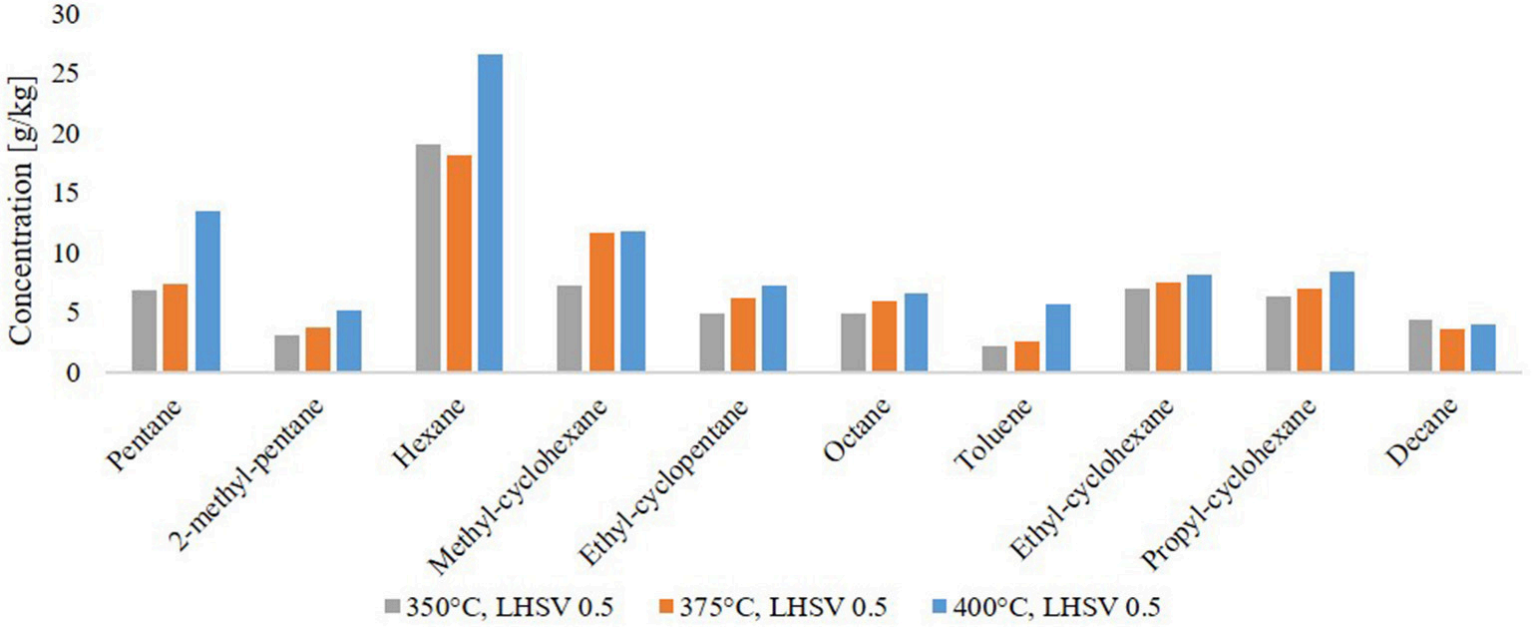
Molecules found in the hydrocarbon product phase by GC-MS analysis.

By GC-MS analysis, the components in the HDO product phases were determined. The 10 most frequent components are shown in Figure 7. Nine of them are alkanes and cycloalkanes, only one of them is an aromatic hydrocarbon, toluene. The components amount between 6.5 and 10 wt.% Together with the high H/C ratio this implies a high grade of saturation in the organic product. In general, the amount of saturated molecules increased with the HDO temperature. This means, that HDO is more effective at higher temperature in the range of 350–400°C. Through the composition of LPP oil, one can assume a few transfer routes from the biomass constituents cellulose, hemicellulose and lignin to the final product after HDO. In LPP oil, the main components were: levoglucosan, 1-(4-hydroxy-3-methoxyphenyl)-2-propanone, 2-hydroxy-3-methyl-2-cyclopentenone, 1-hydroxy-2-butanone, 1-hydroxypropanone, acetic acid and methyl acetate. After fractionation of lignin during pyrolysis, the phenol-alcohols are possibly transformed into cyclohexanes during HDO. This might explain the presence of propyl cyclohexane, as it could be derived from 1-(4-hydroxy-3-methoxyphenyl)-2-propanone, and cyclohexane from guaiacol. Hexane can both be derived from lignin derivatives, such as 2-hydroxy-3-methyl-2-cyclopentenone or levoglucosan, or the cellulose derivative glucose. Pentane is a characteristic hemicellulose fragment, referring to the high amount of pentoses present in hemicellulose (Collard and Blin, 2014).

These suggested pathways are supplemented by many other routes, such as the fractionation from higher molecular structures and formation of C-C bonds, occurring during the pyrolysis of lignocellulosic biomass.

#### Gas phase composition

**Figure 8:**
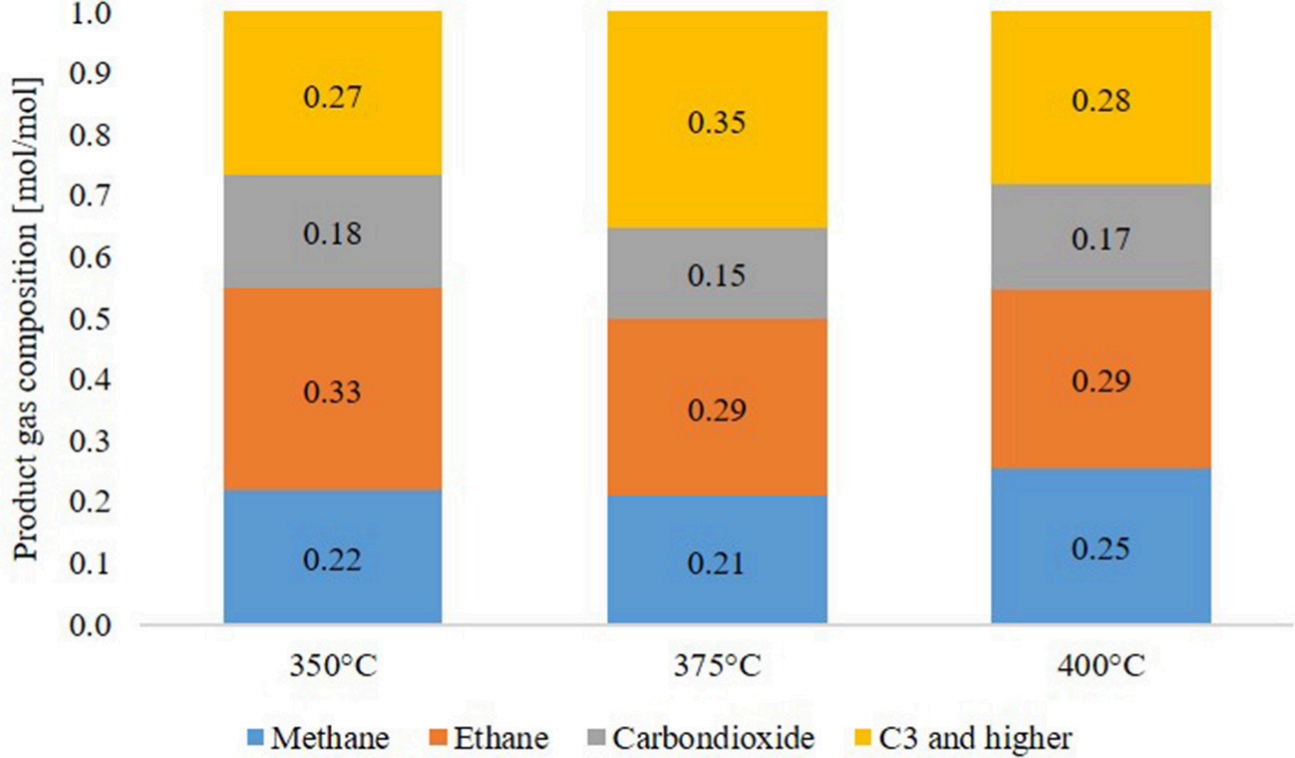
Product gas composition after 12 h of experiment.

The main components of the product gas phase, given in Figure 8, were alkanes as methane and ethane. No oxygen or nitrogen was detected. Acetylene was measured but only detected in the first few hours of experiments as a startup effect. The rest of the gas phase was assumed to be “C_3_ and higher,” describing all alkanes and alkenes with 3 or more carbon atoms. Due to the high excess, the gas phase consisted to about 95 mol% of hydrogen and only to about 5 mol% of product gas. Although little differences are visible, no temperature dependency was detected. Cracking reactions start at elevated temperature and are not observable in large amounts at those process conditions.

## Discussion

HDO has been performed successfully in a single-step process. The water content of LPP oil could be decreased to below 0.05 wt.%. The carbon loss into the aqueous product phase was very low, with carbon contents of 0.5 wt.% at 400°C. Nine of the 10 most frequent components were alkanes and cycloalkanes, as determined by GC-MS analysis. Decreasing HDO rate at 350°C indicates deactivation of the catalyst. The carbon transfer from LPP oil into the hydrocarbon product phase was highest at 400°C. Also the H/C ratio, an indicator for the degree and effectiveness of HDO, was highest at this temperature. The decreasing H/C ratio even at 400°C indicates catalyst activation loss. This can be caused by coke formation, another potential reason might be sulfur depletion during HDO. Ongoing tests indicate a more stable operation if 1000 ppm of sulfur are added to LPP oil (Treusch et al., 2018).

Coke formation at HDO of LPP oil was very low with about 1.35 wt.% based on LPP oil and H_2_ feed and didn't depend on the temperature at the conditions mentioned above. Assuming that the mass growth at the catalyst was 100 wt.% carbon, this still results in a comparable very low carbon transfer from LPP oil to coke of 7.5 wt.%, despite the high LHSV. In comparison, Kim G. et al. proposed a two-step process at an overall LHSV of 0.4 h^−1^, obtaining 1–17 g coke per g pyrolysis oil in the first step at 100–190°C and 1–23 g coke per g pyrolysis oil in the second step at 300–390°C (Kim G. et al., 2017). These results are most likely to be effected by the pyrolysis oil itself. Plugs are typically polymerized bio-oil and inorganic constituents (Olarte et al., 2016). Additionally, organic condensation products of partially upgraded pyrolysis oil components lead to fouling of the catalyst, inhibiting educts to bind to the catalyst and get hydrogenated, which leads to more coking (De Miguel Mercader et al., 2011; Weber et al., 2015). Another point is coke, that is already contained in pyrolysis oil. Fast pyrolysis oils usually contain between 0.3 and 3 wt.% particles (Bridgwater and Peacocke, 2000). In LPP oil, no particles were detected as they are retained by the heat carrier oil during the liquid phase pyrolysis step. Furthermore, through the high dilution by water, heat of reaction is buffered and coke formation, caused by overheating of the catalyst surface, is lowered. At the relatively high LHSV of 0.5 h^−1^, the temperature profile in the reactor shows a lower temperature at the top due to the high heat capacity of water. This results in a short preheating zone and might explain the low coke formation, as high heating ramps promote coking (De Miguel Mercader et al., 2011). Water is also described as stabilization agent for instable charged molecules in pyrolysis oil, reducing the activation energy of ketonisation and increasing the driving force for forming ketones, that are afterwards hydrodeoxygenated (De Miguel Mercader et al., 2011). These reactions usually occur at the front end of the reactor, where coke formation is highest (Elliott et al., 2009).

Compared to LPP oil, the water content of fast pyrolysis oils is much lower. HDO reactions are highly exothermic. Therefore, a two-step process is necessary, where the first step acts as a stabilizing step. It reduces the reactivity of functional groups such as aldehydes, ketones and double C-C bonds (Laurent et al., 1992). Routray et al. described the goal of the first, mild HDO step to be the reduction of some more active compounds like alkenes, aromatics and carbonyl groups, as they are most likely responsible for coke formation. They proposed a two-step process with mild hydrotreatment taking place at 130°C using a Ru/C catalyst and deep HDO taking place at 300–400°C using a Pt/ZrP catalyst. Both steps were performed at 140–150 bar. Although they managed to produce a hydrocarbon phase with primarily cyclic alkanes, after 55 h time on stream (TOS), more than 25 wt.% of the carbon contained in the feed pyrolysis oil was transformed into coke. Plugging by coke formation occurred in all experiments after 55–72 h TOS. (Routray et al., 2017) Elliott et al. described coking in single step processes at 340°C after 30–40 h TOS (Elliott et al., 2009). Olarte et al. observed plugging of the reactor in a single-step reference experiment using fast pyrolysis oil after 48 h TOS at a space velocity of 0.1 h^−1^ (ml_oil_/ml_catalyst_) (Olarte et al., 2016). Kim I. et al. investigated a preceding extraction step to remove particles and most likely lignin components, which are partly responsible for coking. Although experiments were performed at high liquid hourly space velocities of up to 2.3 h^−1^, they observed rapidly decreasing product quality, beginning at about 3 h TOS, resulting in a product with 6.1 wt.% oxygen after 13.1 h TOS. Due to plugging, experiments had to be stopped after 5.7–14.2 h TOS (Kim I. et al., 2017).

The low coke formation during HDO is significant for LPP oil and distinguishes LPP oil from fast pyrolysis oils. Therefore, a two-step process is not obligatory.

## Author contributions

KT was responsible for laboratory experiments, analytics, data analysis and drafted the manuscript. NS was head of this project at Graz, University of Technology, coordinated the study, was responsible for laboratory experiments and was involved in the conception of the laboratory setup. KS and RN participated in laboratory experiments, analytics and data analysis. PP was head of this project on the site of BDI—BioEnergy International GmbH and coordinated the study. MS is the director of the Institute of Chemical Engineering and Environmental Technology and helped draft the manuscript. All authors gave final approval for publication.

### Conflict of interest statement

The authors declare that the research was conducted in the absence of any commercial or financial relationships that could be construed as a potential conflict of interest.
